# Safety and Efficacy of the Superior Gluteal Artery Perforator (SGAP) Flap in Autologous Breast Reconstruction: Systematic Review and Meta-Analysis

**DOI:** 10.3390/cancers14184420

**Published:** 2022-09-11

**Authors:** Jérôme Martineau, Daniel F. Kalbermatten, Carlo M. Oranges

**Affiliations:** Department of Plastic, Reconstructive and Aesthetic Surgery, Geneva University Hospitals, Geneva University, 1205 Geneva, Switzerland

**Keywords:** SGAP, breast reconstruction, microsurgery, free flap, systematic review, meta-analysis, outcomes

## Abstract

**Simple Summary:**

Breast reconstruction following mastectomy is associated with higher patient satisfaction and quality of life. Autologous breast reconstruction has become increasingly popular in recent decades and offers good long-term results. While the abdomen is typically chosen as the donor site for autologous breast reconstruction, it can be unsuitable for some patients. In this setting, different donor sites, such as the buttocks, can be used to reconstruct the breast. The superior gluteal artery perforator (SGAP) flap is a safe alternative to the deep inferior epigastric artery perforator (DIEP) flap and provides good esthetic results, making it a valuable option for breast cancer patients desiring a postmastectomy autologous breast reconstruction.

**Abstract:**

(1) Background: The superior gluteal artery perforator (SGAP) flap is a prominent technique for autologous breast reconstruction. Unlike other commonly used techniques, current literature on the safety and efficacy of the SGAP flap is heterogenous and limited. The aim of this article was to perform a systematic literature review and meta-analysis of postoperative outcomes and complications associated with SGAP flap autologous breast reconstructions. (2) Methods: A systematic literature search of multiple databases was performed using the PRISMA guidelines. We included articles evaluating SGAP flaps in autologous breast reconstruction. Outcomes and complications were recorded and analyzed. Proportions and their 95% confidence intervals (CIs) were calculated in a random-effects meta-analysis. (3) Results: Fourteen studies meeting inclusion criteria, representing a total of 667 SGAP flaps, were included. The total flap loss rate was 1% (95% CI 0–3%), partial flap loss rate was 1% (95% CI 0–3%), hematoma rate was 3% (95% CI 1–6%), emergent surgical re-exploration rate was 5% (95% CI 2–9%), and overall donor-site complications were 12% (95% CI 4–23%). (4) Conclusions: This systematic review and meta-analysis provide comprehensive knowledge on the efficacy and safety of the SGAP flap in autologous breast reconstruction. It demonstrates its overall safety and low complication rate, validating its important role as an effective option in breast reconstruction.

## 1. Introduction

Breast reconstruction following mastectomy is of great importance for women’s body image, sexuality, and the overall quality of life of cancer patients [1,2,3].

Reconstruction with autologous tissue is associated with numerous advantages compared to implant-based reconstruction, including better esthetic results, more natural breast shape, appearance improvement over time, and fewer overall complications [4,5,6,7]. Most autologous breast reconstructions are based on the use of abdominal tissue as the donor site of flaps, which have proven to be reliable and are associated with good outcomes and high patient satisfaction [8,9,10].

Several alternative donor sites for autologous breast reconstruction exist for patients whose abdominal tissue is unsuitable for reconstruction, oftentimes because of a thin abdomen or previous abdominal surgeries. This includes the back, thigh, and buttocks [11].

Among the alternative options, the use of a superior gluteal artery perforator flap (SGAP) in autologous breast reconstruction is gaining increasing popularity among plastic and reconstructive surgeons. First introduced by Allen and Tucker in 1995 [10], the SGAP flap has since been used as a first-line alternative by several groups when the abdomen is unsuitable as a donor site [12,13,14].

While many studies report on the use of SGAP flaps in autologous breast reconstruction, the current literature on the outcomes and complications is limited by the lack of a comprehensive analysis of available data. The aim of this article was to perform a systematic review and meta-analysis of postoperative outcomes and complications associated with SGAP flaps in breast reconstruction.

## 2. Materials and Methods

### 2.1. Literature Search Methodology

A systematic literature search of English databases, including PubMed, Cochrane Library, and Web of Science, was performed on 25 May 2022, according to the PRISMA guidelines [15]. Our search strings used the following keywords: SGAP, flap, free flap, breast reconstruction, and breast. Our systematic review and meta-analysis was registered on Research Registry (https://www.researchregistry.com/, accessed on 6 August 2022) and was given the registration UIN “reviewregistry1438”.

### 2.2. Selection Criteria

All clinical studies and case series in English that evaluate autologous SGAP flaps in autologous breast reconstruction and report on total flap loss as primary outcome were included in our analysis. Non-English studies, cadaveric and animal studies, studies on other reconstruction techniques, studies that did not report on total and partial flap loss, reviews and meta-analyses, isolated abstracts, and case reports with <2 patients were excluded (Table 1). Studies without clear presentation of outcomes were excluded from the analysis.

### 2.3. Data Extraction

Relevant articles identified from the database search were independently scrutinized by title and abstract by two reviewers (J.M. and C.M.O.) based on the predefined inclusion and exclusion criteria using the JBI Sumari software for systematic review (System for the Unified Management, Assessment, and Review of Information (SUMARI), Joanna Briggs Institute, University of Adelaide, North Adelaide, Adelaide, Australia). In the event of a disagreement, the article was screened by a third reviewer (D.F.K.), and a decision was made after discussion between the three authors. Full texts were then read, and eligibility was assessed independently by two authors (J.M.; C.M.O.). Data from the studies deemed suitable for inclusion were then extracted by two authors (J.M. and C.M.O) into a standardized spreadsheet file. First author, publication year, study period, study design, number of patients, number of flaps, mean age, mean body mass index (BMI) and postoperative outcomes/complications, flap total and partial loss, hematoma, infection, recipient, and donor-site complications and need for emergent surgical re-exploration were collected and included in an Excel file (version 16.30, Microsoft Corp., Redmond, WA, USA).

### 2.4. Outcomes

Primary outcome was defined as flap total loss rate. Secondary outcomes included partial flap necrosis rate, hematoma rate, seroma rate, infection rate, emergent surgical re-exploration, overall donor-site complication, and overall complication rate. Outcomes that were included in the meta-analysis needed to be reported in at least three studies.

### 2.5. Statistical Analysis

Results are expressed as a proportion of a given event on a per-flap basis along with their 95% confidence intervals (CIs) and were calculated in random-effects meta-anlysis using the DerSimonian–Laird model. The heterogeneity between studies was evaluated with the I^2^ statistic along with the Q-statistic *p*-value, with I^2^ with values below 30% considered as low heterogeneity and over 70% as significant heterogeneity [16]. *p*-values < 0.05 were considered as significant heterogeneity. Pooled results were then graphically illustrated in forest plots. Statistical analysis was performed using R version 4.2.1 (R Foundation for Statistical Computing, Vienna, Austria) and its meta-package [17].

## 3. Results

**Figure 1:**
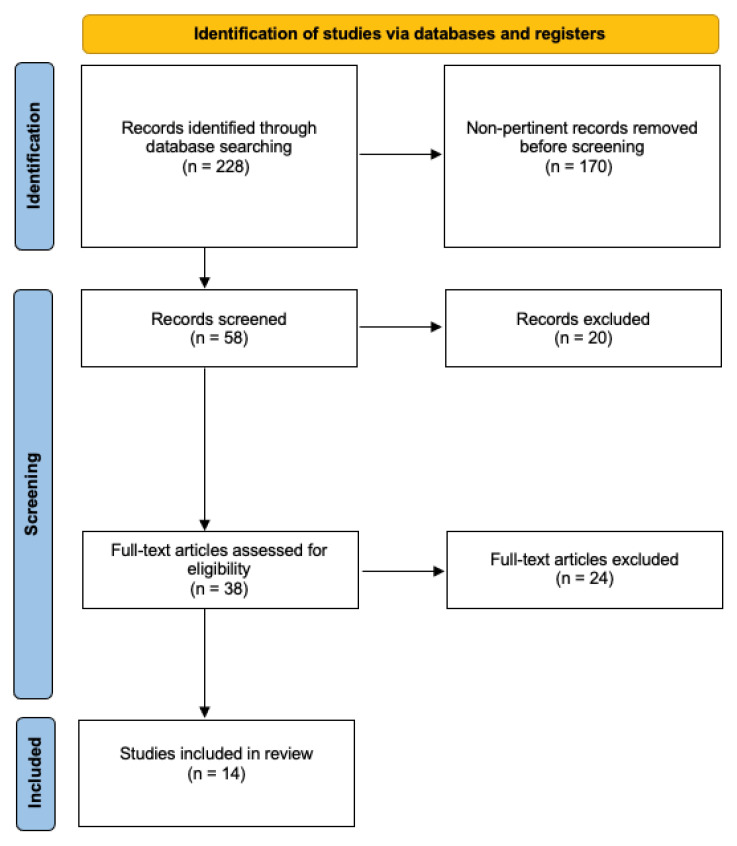
Systematic review flow chart.

Two hundred twenty-eight studies were identified through database searches. After excluding duplicates and screening titles and abstracts, thirty-eight studies were fully read for eligibility assessment, yielding a total of fourteen studies that fully met the inclusion criteria [13,14,18,19,20,21,22,23,24,25,26,27,28,29] (Figure 1).

The fourteen studies included (Table 2) were monocentric studies of retrospective nature. Our meta-analysis covered studies that reported a total of 667 SGAP flaps in 538 patients. Eight of the studies were conducted in the United States of America, and the other six studies were conducted in Europe. Three studies were of comparative nature [22,25,29], and four studies only included patients undergoing bilateral SGAP procedures [19,20,21,27]. Mean age was reported in thirteen studies and ranged from 41 to 52 years. Mean BMI was reported in ten studies and ranged from 20 to 26 kg/m^2^.

**Figure 2:**
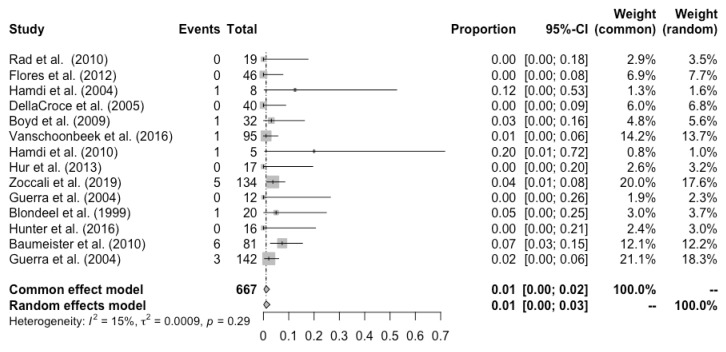
Forest plot of pooled SGAP total flap loss rate after surgery [13,14,18,19,20,21,22,23,24,25,26,27,28,29].

**Figure 3:**
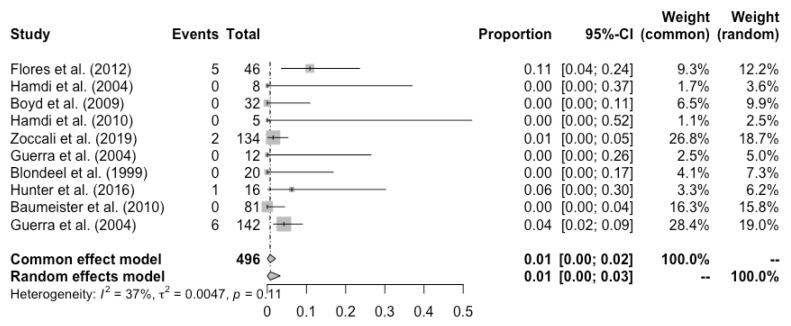
Forest plot of pooled SGAP partial flap necrosis rate after surgery, [13,14,19,20,22,24,26,27,28,29].

**Figure 4:**
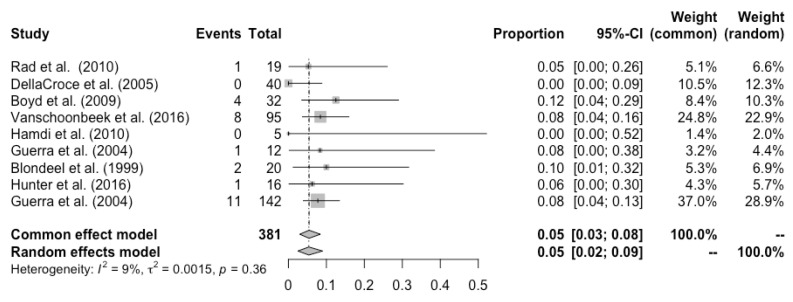
Forest plot of pooled emergent surgical re-exploration rate, [14,18,21,22,23,24,27,28,29].

**Figure 5:**
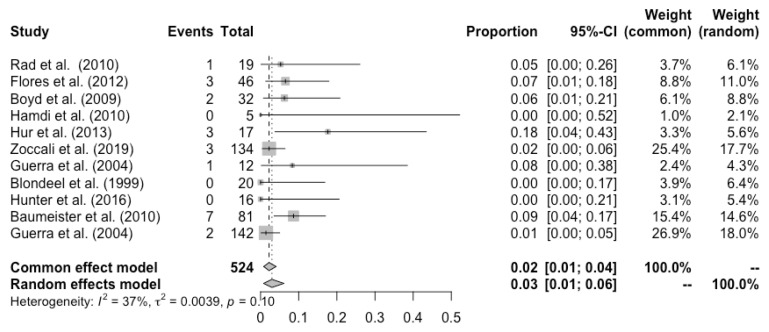
Forest plot of pooled hematoma rate after surgery, [13,14,18,19,22,24,25,26,27,28,29].

**Figure 6:**
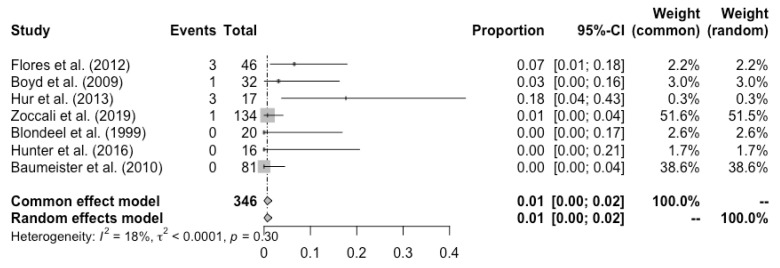
Forest plot of pooled infection rate after surgery, [13,19,22,25,26,28,29].

**Figure 7:**
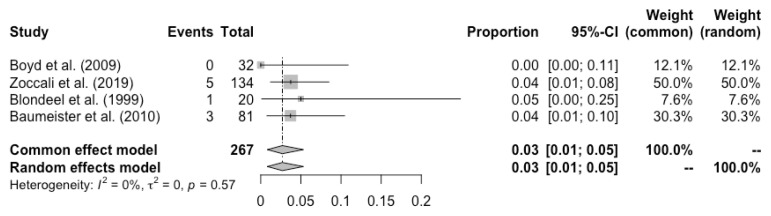
Forest plot of pooled fat necrosis rate after surgery, [13,22,26,28].

**Figure 8:**
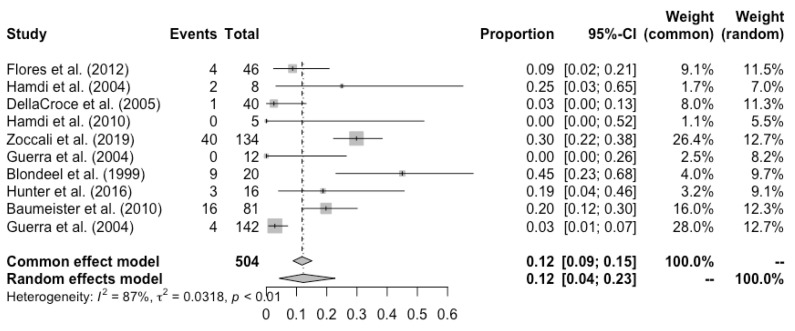
Forest plot of pooled donor-site complication rate after surgery, [13,14,19,20,21,24,26,27,28,29].

**Figure 9:**
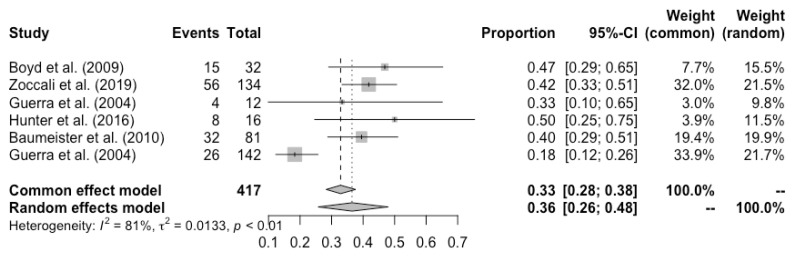
Forest plot of pooled overall complication rate after surgery, [13,14,22,26,27,29].

Complete flap loss was reported in all the studies, and the pooled total flap loss rate was 1% (95% CI: 0–3%) (Figure 2). Partial flap necrosis was reported in 10 studies, and the pooled partial necrosis rate was 1% (95% CI: 0–3%) (Figure 3) [13,14,19,20,22,24,26,27,28,29]. Nine studies reported the emergent surgical re-exploration rate, and the pooled rate was 5% (95% CI: 2–9%) (Figure 4) [14,18,21,22,23,24,27,28,29]. Hematoma rate was reported in eleven studies, with a pooled rate of 3% (95% CI: 1–4%) (Figure 5) [13,14,18,19,22,24,25,26,27,28,29]. Infection rate was reported in seven studies, and the pooled rate was 1% (95% CI: 0–2%) (Figure 6) [13,19,22,25,26,28,29]. Fat necrosis was reported in four studies, and the pooled rate was 3% (95% CI: 1–5%) (Figure 7) [13,22,26,28]. Donor-site complications were reported in ten studies, and the pooled donor-site complication rate was 12% (95% CI: 4–23%) (Figure 8) [13,14,19,20,21,24,26,27,28,29]. Six studies disclosed the overall complication rate, with a pooled rate of 36% (95% CI: 26–48%) (Figure 9) [13,14,22,26,27,29]. Heterogeneity across studies was generally low (I^2^ < 30%); however, it was significant (I^2^ > 70%) for both the donor-site complication rate and overall complication rate.

## 4. Discussion

This study is, to our knowledge, the first systematic review and proportional meta-analysis evaluating the safety and efficacy of the SGAP flap.

It demonstrates that the SGAP flap is safe, with total and partial flap loss rates comparable to other types of flaps used in breast reconstruction, thus implying a reliable blood supply of the flap. Indeed, Ochoa et al. reported a flap failure rate of 1% in their retrospective analysis of 639 DIEP flaps [30]. Gill et al. reported a flap failure rate of 0.5% and a partial flap failure rate of 2.5% in their study of 758 DIEP flaps [31]. Chang et al. reported a total flap loss rate of 0.9% and a partial flap loss rate of 1.4% in their study on 936 breast reconstructions with free TRAM flaps [32]. Moreover, a recent proportional meta-analysis of breast reconstruction using a profunda artery perforator (PAP) flap and transverse musculocutaneous gracilis (TMG) flap also showed pooled total flap loss and partial flap loss rates comparable to our study [33,34].

In the present meta-analysis, two of the included studies compared SGAP and DIEP flaps: Hur et al. investigated the functional outcomes following SGAP flaps and compared them with patients who had undergone breast reconstruction with DIEP flaps. They noticed that, while there were no significant differences between groups in the overall Lower Extremity Functional Score, 11/20 specific items in the score were statistically significantly different between groups—leading them to conclude that patients benefiting from an SGAP flap are more prone to lower extremity fatigue and pain, especially when the activity is more intense. However, their study found no statistical differences in terms of complications between groups.

Flores et al. mention a mild/moderate gait abnormality in two patients following surgery that quickly improved, albeit with intensive physical therapy [19].

Hunter et al. also compared patients who underwent breast reconstruction with SGAP flaps against patients who benefited from DIEP flaps reconstruction and found no statistical differences in terms of complications between groups. They reported a statistical trend (*p* = 0.08) towards longer operative times in SGAP cases.

Unfortunately, the overall complication rate and donor-site complications were not reported on a consistent basis across studies. The pooled overall complication rate was 36% (95% CI: 26–48%), higher than the 23% overall complication rate reported by Quian et al. [33] in their systematic review of PAP flaps and in line with the 30% rate reported by Gill et al. [31] in their DIEP study. The pooled donor-site complication rate was 12% (95% CI: 4–23%), which compares favorably to the DIEP donor-site complication rate of 26% reported by Ochoa et al. [30] and is similar to the DIEP donor-site complication rate of 14% reported by Gill et al. [31] and the TRAM donor-site complication rate of 15% reported by Chang et al. [32]. In our pooled analysis of donor-site complications and overall complications, heterogeneity was significant and is probably related to the lack of standardization in the reporting of outcomes across studies. Noteworthily, there was a large gap between donor-site seroma rates reported in the included studies: Blondeel [28] reported donor-site seroma in 35% of cases, Baumeister et al. [13] and Zoccali et al. [26] reported a seroma rate at the donor site of 14% and 9% respectively, while Guerra et al. reported a donor-site seroma rate of 2% [14], causing us to hypothesize that the definitions of complications changed between authors, leading to a potential under- or overestimation of the donor-site complication and overall complication rate. In the current study, the pooled hematoma rate was quite low at 3% (95% CI: 1–4%), and the pooled infection rate was 1% (95% CI: 0–2%), in line with rates reported in other techniques [30,31,32,33,34], while the pooled fat necrosis rate was at 3% (95% CI: 1–5%)—somewhat lower than the 12.9% and 10.4% rate reported in Gill et al. and Ochoa et al.’s DIEP retrospective studies.

While most authors define the abdominal tissue as being the preferred donor site, some mention that the SGAP flap can also represent an adequate first-line option in autologous breast reconstruction [12,29]. Nevertheless, most of the studies included in this systematic review and meta-analysis consider the SGAP flap as an alternative to the DIEP flap when the amount of abdominal tissue is insufficient or for patients with previous abdominal surgery.

In terms of esthetic results, the authors note that patients are satisfied with the scars being hidden by normal underwear, and Yaghoubian et al. mention that the lack of “mirror visibility” of the scar diminishes patients’ awareness of it [10,12,28,29]. Authors also observed that good breast projection and natural breast shape are obtained with SGAP flap reconstruction [13,18,27]. Guerra et al. explain that the fact that the gluteal fat is somewhat more rigid than the abdominal fat may lead to firmer and more projected breasts [14]. Most authors agree that esthetic outcomes are generally excellent. Rad et al. mention that donor-site contour deformity is sometimes bothersome to patients and note that there is a frequent need for secondary donor-site revision procedures [18]. Zoccali et al. report a contour deformity at the donor site in 9.7% of their cases, with 10% of the patients benefiting from revision surgery in this context [26]. In a large series of 142 cases, Guerra et al. report a donor contour deformity of just 4% and mention that only 6 patients requested a revision of the donor site [14]. Blondeel reports an extremely low donor-site morbidity [28]. Regarding the breast flap, Zoccali et al. report a 15.8% rate of flap-reshaping procedures with liposuction and lipofilling [26]. Unfortunately, none of the studies in this meta-analysis included patient-reported outcomes on esthetic satisfaction and overall satisfaction.

A retrospective comparative study comparing the SGAP, LAP, and DIEP flaps in autologous breast reconstruction using the BREAST-Q score completed by Opsomer et al. showed that patients undergoing SGAP flap breast reconstruction had a similar satisfaction with the appearance of their breasts and a similar outcome satisfaction when compared to patients who underwent DIEP flap reconstruction [35]. However, the satisfaction with the donor site appearance in patients in the SGAP group was tendentially inferior, albeit not statistically significant (*p* = 0.061) compared to the DIEP group.

Commonly highlighted drawbacks of the SGAP flap are its technical difficulty associated with a long intramuscular dissection of the pedicle and harvesting of the flap, required intra-operative position changes, and a longer operative time [13,19,21,26,27,29]. Interestingly, Guerra et al. reported a reduction in operative time over time as their experience with the procedure grew [27].

The SGAP flap vascular anatomy was reliable, with an adequate pedicle size, which varied across studies. Indeed, Blondeel notes that the average pedicle size was 7.8 cm (range: 6–10.5 cm) [28]. Zoccali et al. reported a pedicle length ranging from 8 to 12 cm in their 134 SGAP flap series, with only 4 cases requiring a vein graft to extend the pedicle and allow it to have greater mobility [26]. Guerra et al. report an average pedicle length of 9.1 cm (range: 7–12 cm), with an average artery diameter of 3.38 mm (range: 2–4.5 mm), with a good vessel match with the internal mammary vessels at the recipient site [14]. Preoperative imaging varied across studies, with Hunter et al. stating that they do not use it before SGAP reconstruction, solely relying on intraoperative Doppler [29]. Flores et al. conversely report the use of 3D computed tomography angiography (CTA) in the preoperative setting and mention that it may decrease the overall incidence of flap failure [19]. Rad et al. also mention the use of preoperative 3D CTA [18]. Zoccali et al. note that the preoperative imaging in SGAP reconstruction is primarily determined by surgeon preference [26]. Interestingly, Zoccali et al. also performed a prospective comparative study on the use of preoperative vascular mapping by magnetic resonance imaging (MRI) compared to the use of Doppler only and found no difference in operative time, complications, and overall outcomes between groups [36].

Rad et al. and Flores et al. both report the use of the lateral septocutaneous superior gluteal artery perforator flap (LSGAP), allowing them to gain more pedicle length and diminishing donor-site contour deformity compared to the classic medial location of the SGAP flap, where central gluteal fat removal can create a deformity [18,19]. Nevertheless, the LSGAP was feasible in only 47% of the patients in the Rad et al. series because of a clear dominance of a central perforator in the rest of the cases.

Most authors describe a flap raise with one perforator; however, Hunter et al., for instance, describe four bipedicled flaps in patients who had two separate, distinct perforators [29]. The SGAP flap volume was described as adequate, with Zoccali et al. [26] reporting a mean flap weight of 465 g (range: 259–568 g), Flores et al. [19] reporting an average flap weight of 570 g (SD 229.6 g), and Guerra et al. [14] reporting an average weight of 451 g (range: 190–894 g). Blondeel describes the flap offered enough bulk to meet the contralateral breast size in all cases, with the biggest flap in his series weighing 760 g [28].

This meta-analysis shows interesting results. Nonetheless, several limitations must be considered. The sample size was relatively limited, and heterogeneity was evidenced. Studies in this meta-analysis did not include patient-reported esthetic outcomes and overall satisfaction or any other objective and subjective outcomes of the esthetic results. Moreover, while we compare our results to large retrospective studies or other meta-analyses of proportions, the better way to compare the SGAP flaps would be through large multi-centric comparative studies or, even more ideally, through a meta-analysis of comparative studies.

## 5. Conclusions

This systematic review and meta-analysis provide comprehensive knowledge of outcomes and complications of the SGAP flap, demonstrating that it is a safe and efficient option for autologous breast reconstruction. While the dissection is technically difficult and the operative times are longer, the overall outcomes and esthetic results are excellent. Larger studies, ideally on functional outcomes and with patient-reported outcomes, are warranted to confirm these findings. All things considered, the SGAP flap is a valuable option when abdominal tissue is unsuitable for autologous reconstruction in breast cancer patients following mastectomy.

## Figures and Tables

**Table 1:** Inclusion/exclusion criteria.

PICOS	Inclusion	Exclusion
Population	Adults undergoing breast reconstruction	Animal, cadaveric studies
	
Intervention	SGAP flap for breast reconstruction	Other flaps
Comparator	N/A	N/A
Outcomes	Primary outcome flap total loss rate, complications	Studies that do not report the primary outcome
Study design	Clinical studies, case series	Reviews, congress abstracts, letters, case reports with <2 patients
	

**Table 2:** Clinical characteristics of the included studies.

Author	Year	Study Period	Patients (n)	Flaps (n)	Types of Flaps	Mean Age (SD or Range)	Mean BMI (SD or Range)
Rad et al. [18]	2010	2008–2009	12	19	9 LSGAP, 10 SGAP	49 (10)	22.8 (3.4)
Flores et al. [19]	2012	2005–2010	23	46	35 SGAP, 11 LSGAP	48 (7.3)	23.8 (3.3)
Hamdi et al. [20]	2004	1996–2002	4	8	8 SGAP	***	***
DellaCroce et al. [21]	2005	N/A	20	40	40 SGAP	43	***
Boyd et al. [22]	2009	2001–2007	25	32	32 SGAP	51	***
Vanschoonbeek et al. [23]	2016	1997–2013	74	95	95 SGAP	42.2 (8.8)	21.5 (3.4)
Hamdi et al. [24]	2010	2002–2009	5	5	5 SGAP	46.4 (3.9)	24.7 (4.3)
Hur et al. [25]	2013	2009–2010	17	17	17 SGAP	46.1 (8.1)	23.1 (3.0)
Zoccali et al. [26]	2019	2009–2017	119	134	134 SGAP	43 (24–63)	25.3 (22–35)
Guerra et al. [27]	2004	1993–2003	6	12	12 SGAP	41	***
Blondeel et al. [28]	1999	1996–1999	16	20	20 SGAP	42.2 (34–56)	20.2 (17.4–23.6)
Hunter et al. [29]	2016	2007–2014	13	16	16 SGAP	52.2 (45–68)	25.5 (19–39)
Baumeister et al. [13]	2010	2002–2008	62	81	81 SGAP	44 (17–65)	22.5
Guerra et al. [14]	2004	1993–2002	142	142	142 SGAP	46 (32–60)	21 (19–24.9)

LSGAP—lateral septocutaneous superior gluteal artery perforator, SGAP—superior gluteal artery perforator, ***—Data not reported in the study.
